# A multimethod synthesis of Covid-19 education research: the tightrope between covidization and meaningfulness

**DOI:** 10.1007/s10209-023-00989-w

**Published:** 2023-03-21

**Authors:** Mohammed Saqr, Miroslava Raspopovic Milic, Katina Pancheva, Jovana Jovic, Elitsa V. Peltekova, Miguel Á. Conde

**Affiliations:** 1 School of Computing, University of Eastern Finland, Joensuu Campus, Yliopistokatu 2, 80100 Joensuu, Finland; 2 Faculty of Information Technology, Belgrade Metropolitan University, Tadeuša Košćuška 63, 11000 Belgrade, Serbia; 3 Sofia University, Sofia, Bulgaria; 4 Department of Information Technologies, Faculty of Mathematics and Informatics, Sofia University St. Kliment Ohridski, Sofia, Bulgaria; 5 Department of Mechanics, Computer Science and Aerospace Engineering, Robotics Group, Universidad de León, Campus de Vegazana S/N, 24071 León, Spain

## Abstract

This study offers a comprehensive analysis of COVID-19 research in education. A multi-methods approach was used to capture the full breadth of educational research. As such, a bibliometric analysis, structural topic modeling, and qualitative synthesis of top papers were combined. A total of 4,201 articles were retrieved from Scopus, mostly published from 2019 to 2021. In this work special attention is paid to analyzing and synthesizing findings about: (i) status of research about COVID-19 regarding frequencies, venues, publishing countries, (ii) identification of main topics in the COVID-19 research, and (iii) identification of the major themes in most cited articles and their impact on the educational community. Structural topic modeling identified three main groups of topics that related to education in general, moving to online education, or diverse topics (e.g., perceptions, inclusion, medical education, engagement and motivation, well-being, and equality). A deeper analysis of the papers that received most attention revealed that problem understanding was the dominating theme of papers, followed by challenges, impact, guidance, online migration, and tools and resources. A vast number of papers were produced. However, thoughtful, well-planned, and meaningful research was hard to conceptualize or implement, and a sense of urgency led to a deluge of research with thin contributions in a time of dire need to genuine insights.

## Introduction

The last months of 2019 have witnessed an unprecedented situation that humanity has not seen in a hundred years. The initial reactions of disbelief, hesitance and denial have wasted precious opportunities to prepare or at least to take much needed calculated steps [1–3]. Perhaps, the way the situation developed at a staggering speed has made planning practically impossible. Universities around the world, in response to the global pandemic, were forced to cancel their face-to-face classes and shift to online education. Such a decision was taken overnight leading students, teachers, and families to a reality they had to accommodate with the wherewithal at hand [1–4].

Online learning has become the crucial tool for the online transition, lectures were delivered though real-time video conferences, e.g., Zoom, Hangouts and Teams [5]. Several other forms were also adopted, e.g., video recordings, asynchronous forum discussions, or messaging through emails [3]. Such rapid changes, in the way learning was delivered, has influenced student satisfaction, mental well-being and a willingness to accept the “new normal” [5]. Teachers had to develop learning materials in new digital forms leading to a large increase in workload and possibly time trying to learn necessary digital skills or use new software [2]. Furthermore, teachers had to develop initiatives that help mitigate the unfolding situation, overcome the limitations of virtual teaching and possibly improve interactions with students [6, 7]. Families had to be involved in the teaching process, facilitate home schooling and help their children with the stressful situation [8]. Universities created—or we better say improvised– guidelines that detail how to respond to emergency in various shapes or forms, e.g., “Emergency Management Plan (EMP)”, “Crisis Management Plan,” or “Business Continuity Plan (BCP)” containing essentially the four phases of emergency management: preparedness, response, recovery and mitigation [9].

The accelerating situation has led to new realities where the educational community needed novel insights about different aspects, e.g., students, teachers, pedagogy, tools, and implementations. Therefore, researchers have been racing to offer their insights regarding their experience, students’ perceptions, tools, and ways to optimize learning and teaching, to mention a few [1, 4, 10]. Funding agencies have also tried to help researchers with fast-track grants targeting education during the pandemic, for instance some Erasmus + calls were launched in 2020. To that end, a large volume of research has been produced across vast and diverse areas that requires a synthesis. In this paper, we take a mixed methods approach combining (1) in-depth qualitative analysis of the top 54 cited papers, (2) bibliometric analysis of the publication meta-data, and (3) Structural Topic Models (STM) to make sense of the large number of publications and compile the published research into “topics” which we analyze and offer a concise analysis of the articles content.

Bibliometric analysis offers an overarching quantitative view of scientific research through the analysis of meta-data [11, 12]. Bibliometrics have been used widely across several fields to map scientific productivity, assess impact, dissemination, collaborative patterns, and research trends [13]. This approach relies on several analytical techniques, e.g., visualization, network analysis and statistical methods. However, bibliometrics is commonly criticized for the lack of qualitative and nuanced analysis [14]. Therefore, we augment our approach with qualitative analysis of the top 54 cited papers as well as STM for the analysis of research themes [15].

Despite the recency of STM as a technique, STM has gained an increasing role as a valuable tool for studying textual data across social sciences [16, 17]. Using STM, researchers are able to “mine” latent (often referred to as hidden) topics automatically from the large corpora of text using “unsupervised methods” [18]. That is, *topics* are inferred from the text without a priori assignment or manual coding of the data into predefined categories (“supervised methods”) [19]. The inferred *topics* represent themes within the dataset that have semantic associations. Two types of models exist, single membership models where each document belongs to a single topic and mixed membership where a document represents a mixture of topics which is used in our study. The use of STM could augment bibliometric analysis through discovery of the research themes and the “hidden topics” [16, 17]. In doing so, STM has an advantage over traditional keyword analysis which are usually dominated by most frequent keywords undermining several important themes within the corpus under study. STM has been used across several studies to reveal predominant research themes, e.g., [20–22].

Few bibliometrics studies have tried to cover research about the pandemic, e.g., [21, 22]. Yet, such studies have focused mostly on online education, used a limited dataset or lacked a nuanced qualitative analysis that synthesizes the results beyond the metrics and indicators, e.g., [23]. Our study aims to bridge such gaps. The research questions of this study are:

- **RQ1:** What is the status of research about COVID-19 regarding frequencies, dissemination venues, and publishing countries?
- **RQ2**: What are the main topics of research in the COVID-19 research and how such topics were discussed?
- **RQ3**: What are the major themes in most cited articles and how such themes have informed the educational community about living with the pandemic?

The rest of the paper is structured as follows: the following section presents the methods employed in the study, followed by a section devoted to detailed description of the obtained results regarding each research question with extensive discussion. Finally, conclusions and remarks are presented in the last section.

## Methods

The search was performed on Scopus database since it has a robust well-curated collection of articles that included almost all of Web of Science with a broader coverage for social science topics relevant to our study [24]. The search keywords were chosen to capture all variations of the Pandemic keyword as well as the education and teaching to reflect the context and therefore we choose the following keywords:

- (*covid* OR covid*19* OR covid*-19* OR CORONA *VIRUS* OR “*SARS-CoV-2*") in the title and keywords of all articles and (“Education*” OR “Teach*”) in title, keywords, and abstracts of all articles.

The search for the pandemic keywords involved only titles, keywords, and Scopus categorized keywords. Several iterations of search with different keywords were assessed, in which a sample of articles were assessed for relevance and accuracy. The final search was decided with consensus among researchers that the keywords bring most relevant results and avoids adding “noise.” A decision was made to exclude abstracts from the search for the pandemic keywords since initial searches with abstracts included a large number of irrelevant articles, and thereupon we decided to include articles which authors explicitly stated COVID-19 (or variations of the keyword) relevance through expressing it in the title or the keywords. On the other hand, the education and teaching keywords were searched in article abstracts, keywords, and titles. The keyword learning was also excluded since it brought lots of irrelevant articles, such as articles related to machine learning. The search was performed on 15^th^ of February 2022 and the meta-data was retrieved, processed, and prepared for analysis.

**To answer RQ1**: Bibliometric analysis was performed using Bibliometrix package [25], which is an open source R package that provides a toolset for analysis of bibliographic meta-data. Frequencies of citations, article statistics and top articles were computed and plotted using R statistical language with the help of Bibliometrix.

**To answer RQ2**: We used structural topic modeling (STM). STM has gained an increasing role as a valuable tool for studying textual data across social sciences [16, 17]. Using STM, researchers are able to “mine” latent (often referred to as hidden) topics automatically from the large corpora of text using “unsupervised methods” [18]. That is, *topics* are inferred from the text without a priori assignment or manual coding of the data into predefined categories (“supervised methods”) [15, 18]. The inferred *topics* represent themes within the dataset that have semantic associations. Two types of models exist, single membership models where each document belongs to a single topic and mixed membership where a document represents a mixture of topics which is used in our study. The use of STM could augment bibliometric analysis through discovery of the research themes and the “hidden topics” [16, 17]. In doing so, STM has an advantage over traditional keyword analysis which are usually dominated by most frequent keywords undermining several important themes within the corpus under study. STM has been used across several studies to reveal predominant research themes, e.g., for the analysis of education technology topics [18].

To identify the main themes of research through structural topic modeling we used R package *stm* which provides methods for probabilistic topic models, STM in our case. A topic is defined as a mixture of words where each word belongs to a topic with a certain probability. A document could have a mixture of topics, i.e., several topics could describe a single document with a certain probability. The *stm* package implements Latent Dirichlet Allocation (LDA) and uses a variational Expectation–Maximization algorithm to estimate the models and their parameters. The topics were modeled using the article's meta-data (title, abstract, keywords) as input [19]. The abstract and title were cleaned from stop words. Since different keywords may represent the same meaning and could result in erroneous results, we performed an exhaustive cleaning process where we combined similar keywords together using Google Openrefine [12, 26]. For instance, Learning Management system, LMS and learning management systems were combined together. The cleaning also removed keywords that are used to indicate COVID-19 (e.g., covid, covid19, covid-19 pandemic, Corona Virus) since they were among our search keywords. The estimation of the topic modeling was performed after the cleaning step.

An essential step of topic modeling is in choosing the number of topics. However, there is no optimum way to identify such numbers [27, 28]. Several methods exist to assist in this process, the most recommended of which are semantic coherence, exclusivity, and human judgment, which we applied in our study [15]. Semantic coherence is a criterion that is maximized when the most probable words co-occur together and correlates with human judgment. Nevertheless, as noticed by [19], semantic coherence is often dominated by frequent and common keywords, e.g., education and students in our case. Therefore, a measure for the specificity and uniqueness of the keyword was conceptualized to better separate different topics. Exclusivity, as the name suggests, reflects how exclusive the word is in a given topic [29]. Semantic coherence and exclusivity, while offering valuable guidance they “offer no particular statistical guarantees and should not be seen as estimating the “true” number of topics” [19], or as a substitute for careful examination, validation and extensive evaluation by human judgment [27]. Therefore, we followed the guidelines by augmenting the statistical parameters with consensus from experts about the most appropriate number of topics.

We estimated 40 models, the smallest of which had five topics and the largest had 45 topics. The semantic coherence and exclusivity were plotted and examined; ten topics had favorable yet close values. The topics were then examined by four experts who had to rank the best number of topics based on the following criteria [15]:

1. the meaningfulness of the topic keywords forming a single theme.
2. no significant overlap with other topics
3. no significant dissonance of the representative words.

Each of the experts judged these criteria and the top three topics were examined, discussed and a consensus was reached among the experts that the number of topics that brings unified themes together, with least overlap and dissonance was sixteen topics.

**To answer RQ3**: The top 70 articles were retrieved according to the number of citations. While our intention was to report on all the 70 articles, we found that some of these articles were very short (less than a full page or just an extended abstract) and had no methods or results sections. Therefore, a quality assessment was performed so that very short articles (single page articles), articles without methods or results section, or articles with very small sample size (e.g., n = 3) were flagged. The quality criteria were agreed by the three researchers and applied to each of the analyzed papers, when a paper was flagged as a candidate to be excluded by one of the researchers the rest of the authors checked it also in order to make the proper decision and reach a census to exclude the paper. A total of 16 articles were rejected based on a consensus of the three authors and meeting the exclusion criteria. The remaining articles were qualitatively coded according to the themes representing the content of these articles by three researchers. The themes were developed using an inductive or grounded theory approach, i.e., developing the themes directly from the articles [28]. Three authors met and coded the articles and reached an agreement after several iterations on the following themes as representative of the main themes in the topics: challenges, guidance, impact, problem understanding, online migration, and tools and resources. In addition, during this classification the target group that the articles were dealing with was also considered, i.e., teachers, students.

## Results and discussion

### RQ1: The status of research about COVID-19

**Fig. 1:**
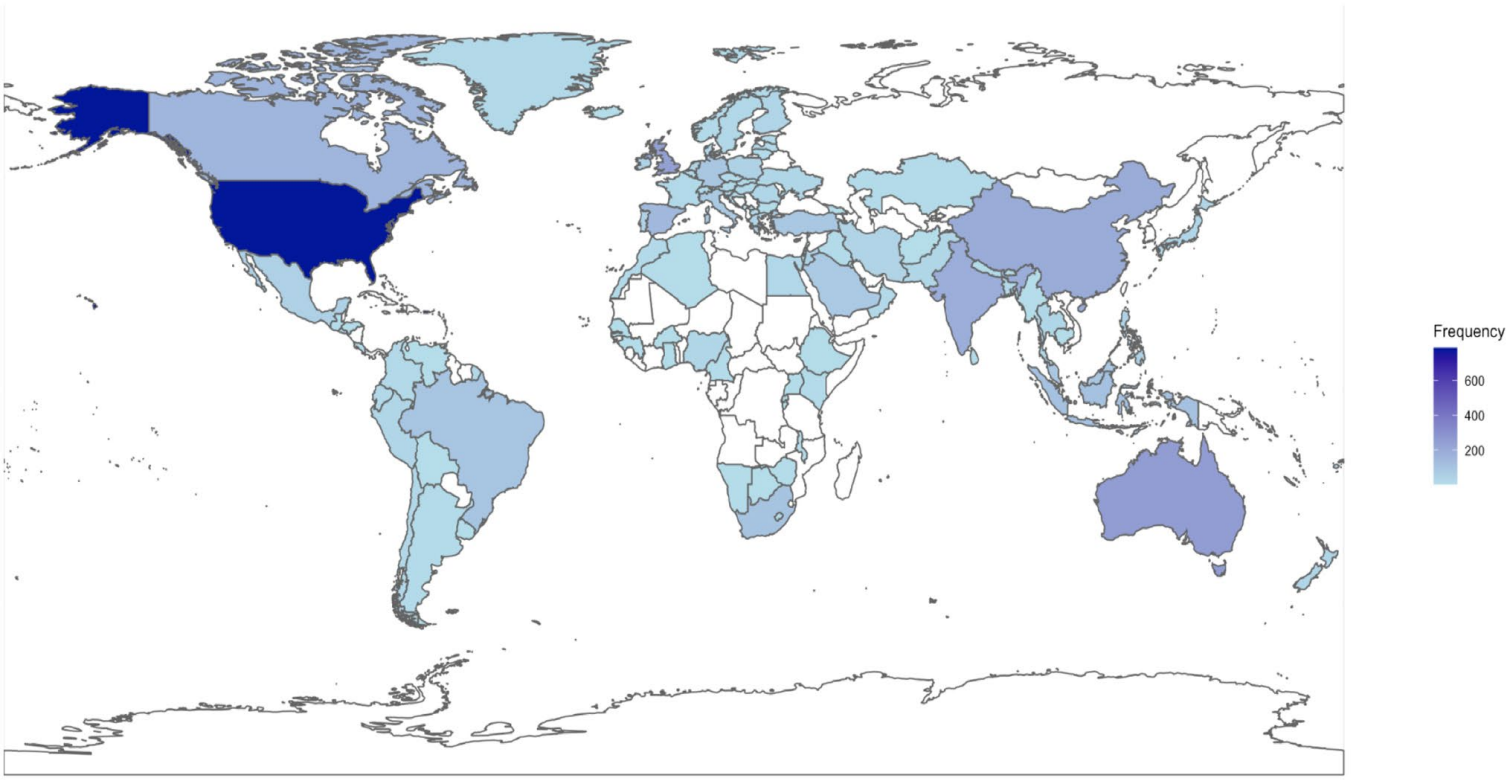
A world map showing the distribution of research productivity by country. Darker colors represent higher research numbers

The dataset included 4,201 articles, most of which were published in 2021. Three articles were published in 2019, 958 (23%) were published in 2020 and 2,861 were published in 2021. Most of the articles were journal articles 3,310 (78%) with a comparatively low number of conference articles 329 (8%). A total of 12,998 authors contributed to those articles and the majority of them appeared only a single time (93%). Most of the articles in our dataset were collaborative with an average of three authors per document. The USA was on the top of our list of most productive countries in terms of number of articles (21), followed by the United Kingdom with around 7, India 5, Spain, China, and Australia with around 4 of the articles (Fig. 1). Yet, the total citations did not mirror the list of top productive countries completely, where we see Spain, Canada in higher positions Table 1.

**Table 1:** Country productivity, citations, and collaboration indicators

Country	*n*	*Percentage*	MCP	Cites	*AC*
United states	572	21.87	9.09	2897	5.07
United Kingdom	190	7.27	20.00	1341	7.06
India	131	5.01	7.63	426	3.25
China	120	4.59	28.33	541	4.51
Australia	118	4.51	21.19	580	4.92
Spain	106	4.05	18.87	920	8.68
Canada	85	3.25	14.12	735	8.65
Malaysia	64	2.45	20.31	207	3.23
Saudi Arabia	62	2.37	12.90	267	4.31
Turkey	59	2.26	8.47	131	2.22
Germany	58	2.22	20.69	330	5.69
South Africa	58	2.22	12.07	175	3.02

n, number of articles; MCP,  % of articles with other countries; AC,  average citations per article

### Publication venues

The dataset contained 1,098 different unique publication venues, i.e., different journals and conferences. Some 553 (49%) of the venues published only a single article about COVID, 196 (18%) published two articles and 104 (9%) published three articles. This diversity in publication venues may reflect the emergent situation where no journals or publications venues were devoted or specialized in such an unprecedented situation. The top publishers in the dataset were open access publishers, some of them have short publication processes [29], and so were the journals that were on the top of our list. *Sustainability* and *Education Sciences* from MDPI (Multidisciplinary Digital Publishing Institute) were the top journals publishing around 7% of all articles and had around 10% of all citations. *Frontiers in Education* published around 2 of all articles and had only 1% of all citations. The rest of the list were dominated by medical education journals, e.g., BMC *Medical Education*, *Medical Science Educator*, *Journal of Dental Education*, *Academic Medicine*. JMIR *Medical Education*, *Advances in Medical Education and Practice* and *Journal of Surgical Education*. The high representation of medical education journals may reflect the fact that medical education involved significant practical work that required students to be in hospitals where the dangers are paramount [6, 30]. Table 2 presents the full list of the top journals that published the papers that were considered in this analysis.

**Table 2:** Statistics of the venues regarding number of articles and citation patterns

Venue	*n*	% articles	*n* Cites	AC	% of Cites
Sustainability	184	4.38	1275	6.93	6.84
Education Sciences	123	2.93	687	5.59	3.69
Frontiers In Education	87	2.07	184	2.11	0.99
BMC Medical Education	73	1.74	533	7.30	2.86
Education And Information Technologies	61	1.45	186	3.05	1.00
Journal Of Chemical Education	55	1.31	402	7.31	2.16
Medical Science Educator	45	1.07	119	2.64	0.64
Prospects	40	0.95	566	14.15	3.04
Journal of Dental Education	36	0.86	400	11.11	2.15
Academic Medicine	30	0.71	154	5.13	0.83
JMIR Medical Education	30	0.71	162	5.40	0.87
Advances In Medical Education and Practice	29	0.69	76	2.62	0.41
EDUCON	28	0.67	16	0.57	0.09
Library Philosophy and Practice	28	0.67	16	0.57	0.09
Journal Of Surgical Education	27	0.64	664	24.59	3.56

n, number of articles; AC, average citations per article, % articles percentage of all articles in the datasets, nCites: number of citations, % of Cites percentage of all citations in the dataset

### RQ2: The main topics of research in the COVID-19 research

**Fig. 2:**
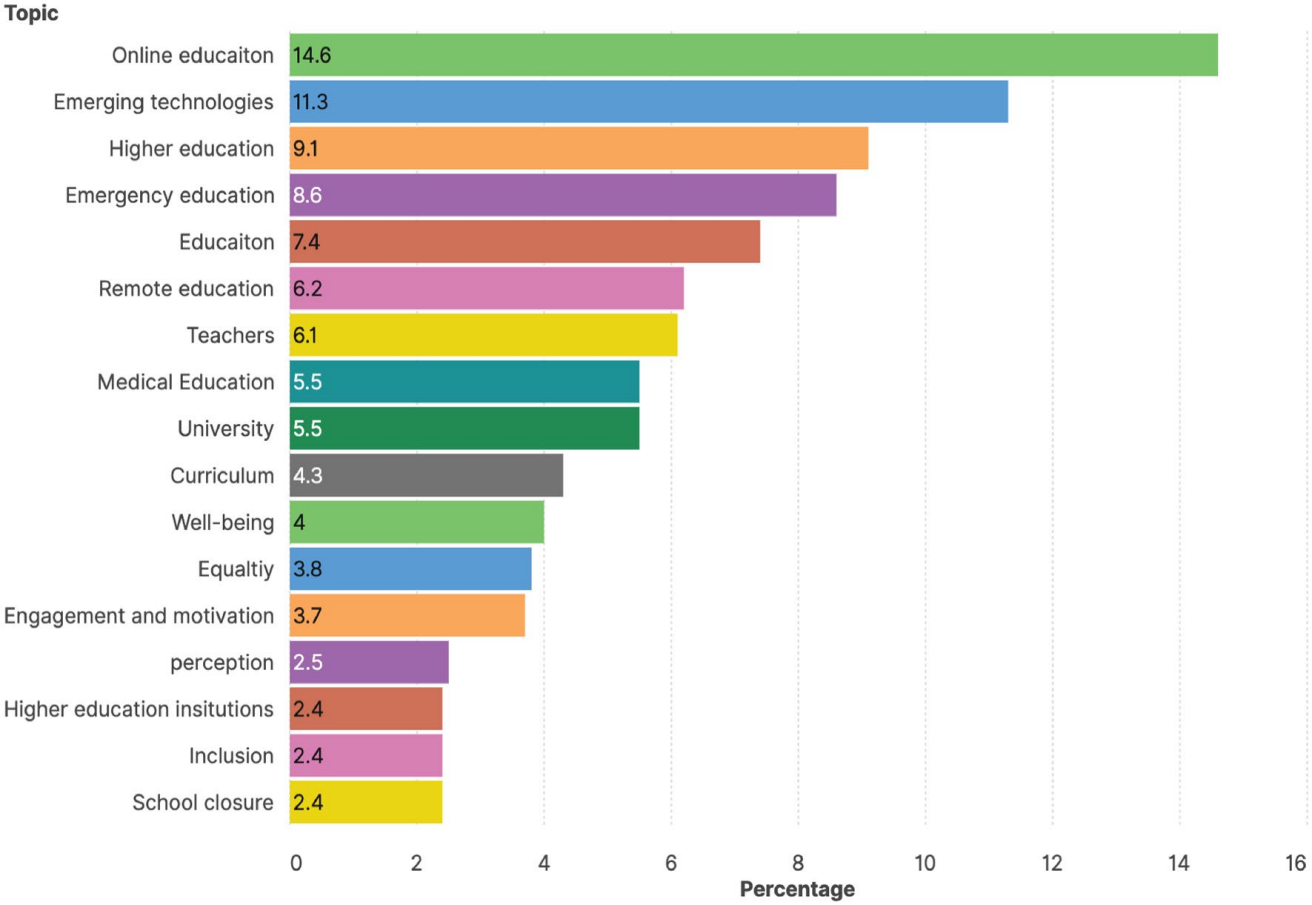
Relative frequency of the sixteen topics in the dataset

A total of 16 topics were identified by the STM, each topic was labeled according to the most probable keywords and the theme representing the topic. The resulting topics were grouped into three main groups of topics: education related, distance/online education and diverse issues. The topics are summarized in detail with the most frequent words in Table 3, Fig. 2. Below is a concise overview with the representative keywords from each topic.

**Table 3:** The topic identified by STM and their characteristic words

Label	Frequent words
University	university, online/distance education, professional development, communication, south Africa, parents, literacy
Higher education	higher education, health, resilience, mental, public, early childhood, educational technology
Education	learning/teaching, sustainability, testing/assessment, environment, mathematics, teaching/learning, multimedia
Curriculum	curriculum, distance education, medical education, online, social distancing, assessment, technology
Higher education institutions	higher education, China, institutions, digital, post-digital, 'new normal', community engagement
*Distance/online education*	
Online education	online/distance education, blended learning, technology, students’ perceptions, evaluation, adaptation, practices
Teachers	teachers, pedagogy, crisis, innovation, blended learning, children, management
Remote education	remote, challenge, schools, educational technology, social work education, post-covid, general public
Emergency education	education, emergency, remote, technology, virtual reality, India, experience
School closure	school closures, physical education, social justice, medicine, self-efficacy, home schooling, medical students
*Diverse issues*	
Perceptions	perceptions, ed-tech, social media, policy, family, descriptive analysis, thematic analysis
Inclusion	inclusion, research, experiences, social, undergraduate, digital competence, qualitative
Medical Education	medical education, technology, simulation, telemedicine, English, culture, strategies
Engagement and motivation	training, student engagement, digital divide, engineering education, survey, motivation, quality
Well-being	well-being, leadership, social media, stress, equity, digitalization, anxiety
Emerging technologies	artificial intelligence, industry 4.0, stem, learning society, lifelong learning, fourth industrial revolution, virtual reality
Equality	inequality, policy, global, students, neo-liberalism, digital literacy, home

Several topics were general, or education related, and these include ***University***, ***Higher education***, ***Education***, ***Higher education institutions***. These topics addressed the broader context of pandemic and education, the role for higher educational institutions, understanding the “new normal” while also thinking of possibilities for a future pandemic or biological hazards and their impact. Several issues were discussed, e.g., internet and infrastructure weaknesses, coping with difficulties, academic career stability, university's financial stability, the complexity of some applied disciplines, student's mental health, costs of fast transformation, and tackling the financial challenges [31, 32].

Distance/online education were among our most discussed topics in the reviewed literature. ***School closure*** was discussed mainly in the context of the effects of the pandemic on learning and teaching [34–35]. Researchers have highlighted the key role of digital teacher competence in transitioning to ***online teaching*** [33] and delivering ***remote education*** as a crisis-response [36]. The crisis situation required an ***emergency*** response for immediate execution [39]. Please see the results of **RQ3** for a more elaborate coverage of online education, guidelines for tackling the pandemic including tools and recommendations.

Several diverse issues have emerged as a reaction to the pandemic. It was obvious that the impact of the pandemic has been unevenly distributed where students with special needs, either physical or psychological needs, were hit the hardest [37, 38], therefore, ***Inclusion and equality*** have been a concern. Many institutions encountered online teaching/learning for the first time where technical infrastructure, quality of the network, computer availability and teachers' competences had a significant role in the successful transition from offline to digital mediums [41]. While digital infrastructure played a vital role in facilitating the transition in developed countries, students and teachers from undeveloped, remote, and rural areas had problems with poor Internet connectivity, network speed or even a lack of electricity [40–41]. Such challenges resulted in a more pronounced impact, lack of equality and inclusion [37, 42].

Students’ ***perception*** of school closures and the large-scale introduction of online learning, in general, was initially positive [43]. However, research revealed that most students had learning barriers as a consequence of the pandemic, despite the introduced transition instructional techniques [44]. Students’ perceptions have also revealed that a mismatch could happen between students’ expectations and teachers' managing online learning [45].

As distance learning prevailed during the times of rising infection rates, students’ ***engagement and motivation*** was an issue. Studying from home required greater self-discipline to follow through with online lessons. On the other hand, lecturers’ unfamiliarity with the new mode of delivery sometimes burdened their students with study materials and assignments. The lack of social interactions, lockdown and restricted physical activities were not easy for the young generations [46]. Such a heavy psychological toll has prompted several scholars to investigate issues of students' ***well-being*** and mental health during the pandemic. Research has shown that most students were under mental and psychological stress [47]. Several articles have investigated the factors that help students handle the unusual situation [48], or how to offer mental and psychological support to students during the pandemic [49]. Such mental support was investigated across all stakeholders, e.g., students, teachers and families [49]. The online transformation of education helped forge a stronger connection between teachers and parents than ever before [50].

***Medical education*** (including dental and nursing education) was among the most discussed topics, and medical education journals dominated the list of our top venues. Several articles discussed the dilemma of the need to train future healthcare professionals in hospitals (where the risk of infection is high) while still protecting the students, teachers and patients [6, 30]. The issue was discussed widely across the world, e.g., in the United Kingdom [51], in China [52] and in the USA. Other articles discussed medical students’ contributions to the delivery of care where healthcare services were restrained [53].

### RQ3: Top cited papers

From the top 70 most cited papers, only 54 passed our quality check and were classified according to 6 different themes: challenges, guidance, impact, problem understanding, online migration, and tools and resources. The category ***problem understanding*** had 26 contributions, which was the highest number of all other themes. ***Challenges*** and ***impact*** had 16 contributions each, while ***guidance***, ***online migration*** and ***tools and resources*** had 14, 13, and 10 contributions, respectively. Table 4 presents most common findings in the analyzed most cited papers for each category.

**Table 4:** The most common findings in top 54 cited studies for each theme

Problem understanding		
Teachers’ issues	Students’ issues	Other issues
There is a need to develop digital competence [5, 33, 55–57] Improve student–teacher interaction [49, 55, 57] Equip the teachers also with socio-affective competences [54] Willingness to change to online and apply new techniques and methodologies [57, 58] Assessment difficulty and need for possible adaptations [58, 59] Importance of making personal connections and increasing student interaction [60] Teaching experience and specialization is very strongly correlated with readiness to distance learning education [61] Teachers’ geographic location is strongly correlated with readiness to adapt to distance learning education [61]	Digital divide: Need of ICT infrastructure [62, 63] sometimes with a high cost [43, 64] Should find ways to cope with stress and anxiety due to the new situation and provide them with tools and experts to support them with the situation [5, 49, 62] Availability ICT for disadvantaged students [49] or for students in different contexts [62, 65, 66] In practical disciplines such as medicine and especially at some moments enhanced virtual curriculum development is required and this could affect the specialty choice [67, 68] Efficiency of live online courses was unsatisfactory among students. However, when live online courses are combined with the flipped classroom it improves [69] Improve communication with teachers and students [56, 69] Higher workload [5, 6, 32, 40, 67]	Need for a pedagogical approach that relies heavily on the social and collaborative components of learning [54] Distance learning is seen as a solution but with the barriers of the need of technological infrastructure and with a high acceptance if there students have a previous experience with it [63] Parents’ anxiety and need to educate from home [35] Mentoring methods are more flexible and sophisticated approaches in order to enhance the potential of new spaces for teaching and learning to teach [70]

Challenges	Impact	Guidance
Teachers and students had to deal with anxiety and frustrations [5, 6, 36, 44, 71] Both students [5, 72] and teachers [4, 50, 73] reported perception of higher workload and lack of computer skills [65] Need for substituting hands-on learning and conducting praxis virtually [6, 63, 70, 73, 74] Many depended on family members to help them to adapt to the online environment [50] Challenge of student retention and student recruitments [4, 50, 75] Lack of motivation and presence of boredom [44, 65, 76] Lack of technical and infrastructural resources [44, 63, 66, 77] Teachers might not be familiar with the process of choosing the most suitable resources [77] Some have questioned whether the digitalization of higher education was too aggressive and if it will leave a negative prejudice on future distance learning [47, 63] Need for complex cognitive and social skills that underpin success in online learning environments [60] Academic performance of students may be affected by socio-demographics [36, 75]	Impact on students’ academic work and personal lives [5, 49, 56, 62, 71, 72] Students’ satisfaction with the role of their university [60, 76, 77] Impact on students’ confidence and their preparedness for the next steps in their studies [51, 67] Students’ perception about the quality and effectiveness of different teaching and learning approaches and experiences [43, 49, 51, 72, 78] Impact on teachers’ planning, teaching, and workload [34, 64] Impact on the digital divide among different socio-demographic communities/groups [5, 62, 79] Impact on the transformation of online education [64, 80, 81] Impact on student interest to study overseas [82] Impact on the readiness of educational institutions for distance education [61]	Adjusting teaching to remote/emergency learning, [55, 70, 83, 84] Posting materials online [3, 66, 73] Providing regular feedback [34, 54, 70, 74] Maintaining online interaction [34, 54, 55, 83, 85] Providing practical training through distance learning [3, 70, 81, 84] Establishing targeted communications for the reassurances of parents and students [3, 34, 84] Making a use of open educational resources and practices [3, 70, 77] Learning how to cope with the stress [86] Using active learning pedagogical approaches, along with simulations and videos [3, 73] Using diversified and individualized assessments [34, 81] Using flexible teaching and assessment methodologies [85] Develop the system for quality assurance of e-learning [79] Establishing support systems for the faculty and students on the institutional level [87]

Online migration		
Interventions	Implications of the change	
Optimism as part of the faculty readiness to the change and willingness in sharing power in the class with students [58] Need to adapt assessment [58, 83] Technological solutions not always driven by best pedagogical practices [88] Possibility to integrate external tools in the institution or to export data to facilitate the use of such tools [36] Necessity to deal with factors such as: technology availability, work at home, heavy workload, digital competence, assessment and supervision and compatibility [36, 50, 83] Necessity to provide the young generation with digital skills and to avoid the different ways of digital divide [50] Online university teaching requires to design activities taking into account the new reality about presence (social, cognitive and facilitatory) [83]	Technical, infrastructural resources and student barriers as key issues for the success of distance learning [63] [65] COVID-19 as a way to launch online learning initiatives at different educational levels [4, 80] Need of professional development designers to overcome the main problems related with the pandemic situation such as equal access to online learning by students and managing demands of the stakeholders in the educational process [4, 58] Emergency remote teaching is not the same as online learning but the experience obtained will lead to a future more sustainable online learning [36] The impact in not only academy but student recruitment, market sustainability, an academic labor-market, and local economies [73]	

Tools and resources		
Tools and resources related to medical education [73, 74] Description about the asynchronous and synchronous tools and approaches followed and lessons learned [76].The type of tools provided by the government in the COVID-19 situation, with access to free educational tools and contents and even using tools not created for educational purposes [80] The importance of using securitization theory during emergency learning [89] The use of open educational resources (OER) and open educational practices (OEP) as effective tools in COVID-19 situation [77] Studies related to tools use and success such as: frameworks to assess educational portal success [79]; adaption of acceptance model to evaluate the acceptance of LMSs [90]; or study of the impact of the pandemic situation in user experience with several educational platforms in China [78] Analysis of the coping strategies with stress levels reported by teachers [86] The use of social media to facilitate interaction between teachers and students in COVID-19 situations and future implications [91]		

The research carried out covers several issues that are related among them. The first one, “problem understanding” was present in most of the evaluated research. The problems were addressed from a local perspective [41, 56, 63, 67, 90], to a global point of view [5], passing through regional or local approaches [31, 33, 47, 49, 54, 55, 58–59, 61, 65–66, 68]. They deal with the stakeholders perspectives [5, 31, 41, 47, 52, 54, 56, 58, 59, 61, 68] and other deals with varied topics related to the context or the conditions in which the activity is carried out [5, 47, 49, 53, 61–66, 90, 91] or the methodologies or solutions employed [52, 55, 58, 63, 67, 68, 86].

Regarding the explored “challenges”, there were great variations. We can summarize them as how COVID-19 requires changes in educational processes [4, 6, 42, 48, 61, 64, 68, 72–73, 75] and how prepared for this are students, teachers, and other stakeholders [4–6, 34, 42, 48, 63, 70–71, 74]. All the stakeholders are required to develop computer skills [63] and need the infrastructure [42, 61, 64, 75] to conduct the teaching and learning in an online way.

The COVID-19 “impacts” were considered at several levels. In the compatibility between the academic and personal life [5, 47, 54, 60, 69, 70], in how teaching is carried out [41, 47, 49, 70, 76] and the associated workload [32, 62], in the transformation of online education [62, 78, 79] and in how universities must adapt to the new context [5] and distance education [59].

Another important issue to be explored in this review is the “online migration.” This issue depends on the context as described above. Some discuss the interventions carried out [42, 55, 58, 74, 92] while others focused more on the implications of this change [4, 34, 56, 61, 63, 78, 81].

Regarding the “guidance” proposed in several cases, some of the guidance was related to teaching materials and methodologies [53, 68, 81, 82], the interaction with students and parents [3, 32, 52, 53, 68, 71, 72, 75, 82–83], ways to provide practical training [3, 68, 79, 82], assessments [32, 79, 83], ways of dealing with the stress [84] and institutional support development [77, 85].

Finally, when exploring the tools and resources used during COVID-19, it is important to take into account that they can depend on the context. For instance, in medical training it could be necessary to take into account how to maintain patient contact, contact with medical experts, develop peer-mentoring techniques, etc. [71, 72]. But in general contexts, the most relevant tools and resources are related to interaction strategies with students [74, 78, 89], the type of resources used during the classes [75], assessment tools [77, 84], and educational platforms [76, 88].

## Reflections and conclusions

We conducted this study with the aim of offering an overarching synthesis of COVID-19 research from the pandemic onslaught till now. A mixed methods approach was used, where we combined quantitative analysis of research productivity with pandemic statistics, structural topic modeling and qualitative synthesis of papers with most attention from the educational community. There are several key findings that warrant reflections.

The analysis has shown that the process of knowledge production about COVID-19 was less skewed compared to educational research in general [12, 93], with a large global participation of 137 different countries in research productivity. Whereas, research was concentrated in large and resourceful countries such as United States [35, 57, 67, 68], China [77], India [49], Germany [33], United Kingdom [51]; we also see several studies that addressed local and non-western contexts, e.g., Philippines [61], Rural South Africa [65], Jordan [63], Romania [56], Indonesia [66]. In fact, a global perspective [46, 64], with wide participation from different countries has helped in understanding the full breadth of impact of the pandemic [39, 41, 63]. In doing so, issues such as inequalities among different students’ subpopulations, as well as disparities in infrastructure and access to internet in, e.g., rural areas, received global attention and were prioritized [37, 42].

Several papers targeted teachers and teacher education [58, 61, 70], others have addressed students [49, 56, 63], yet, very few have researched the perspective of the families, despite that families were heavily involved in the process [50, 68]. Notable also that research was rather skewed toward some research fields, where medical [51, 63, 67, 68], engineering and mathematics education [45, 66, 92] received significant attention from researchers. A finding that could be explained by the idea that such disciplines may require practical face-to-face teaching which was an issue of concern during the pandemic [6, 30].

School closure, the consequences, and the alternative solutions occupied the public discourse as well as the research communities. Yet, schools have gone through several stages. Initially, many countries rushed to school closure which peaked around April 2020. About 1.3 billion students (81.8% of all enrolled) were instructed to stay home; a year later, where the pandemic was more rampant, school closure affected only 12.7% of students, reaching 2.7% as per the last recording in February 2022. Perhaps, the loss of learning time, the heavy toll on learners’ well-being as well as the remarkable burden vulnerable students had to endure [8, 48], has led to a policy where schools “were last to close and first to open” to avoid what the UNESCO called “a generational catastrophe.” Such a potential catastrophe would have resulted in stark inequalities of learning opportunities but also other aspects that school provides, e.g., school meals, physical activities and social interaction [37, 40–41]. Of course, such decisions were aided by prioritizing teacher vaccination, health measures and infection tracing [39, 47].

If anything the pandemic is known for, it is the “impact,” an issue that has been studied from all points of views and perspectives. Therefore, researchers intensively studied the impact of pandemic on workload [34], academic work and personal lives [5, 56], student satisfaction [4, 76], confidence [51], quality of teaching and learning [43, 51], and on vulnerable groups [5, 62]. The impact on mental health and well-being has been a central theme in the pandemic research [8, 47, 49]. Along with the impact, came a long list of articles of recommendations and guidance regarding how to mitigate the impact, or address the challenges. For instance, we saw discussion about technical infrastructure [33, 40], online learning initiatives [4, 80], and sustainable online learning [36].

The rush to move online was accompanied by an accelerating stream of articles about the pandemic [94]. Thoughtful, well-planned, and meaningful research was hard to conceptualize or implement, and a sense of urgency led to a deluge of research with thin contributions in a time of dire need to genuine insights [94–96]. Perhaps, as it has been argued by [94, 97], some may have found an opportune time to jump on the bandwagon of COVID-19 and the possibilities for research funding to capitalize on the need for research about the pandemic, a phenomenon that later became known as Covidization of research [94, 97].

We have used two methods for the analysis: STM and thematic analysis of the top cited papers. While STM is well-established for summarizing the general themes of a large textual dataset, such summarizing power should not be confused with retrieving the “true” content of the documents. As [27] points out, automated text analysis should not substitute careful and thoughtful text examination. Therefore, such methods are “best thought of as amplifying and augmenting careful reading and thoughtful analysis” [27]. Thereupon, a qualitative thematic analysis was performed, which revealed related but also varying themes. Of those themes, some may be hard to pick with a summarizing automatic text analysis, e.g., problem understanding, implications of the change and challenges. As such, we suggest that a careful qualitative analysis may be helpful to draw the full picture of text analysis.

Our article is not without limitations. Our search using keywords—which is the standard in all systematic reviews and bibliometrics—may have missed some articles that did not explicitly mention the pandemic keywords. Our results should not be viewed as encompassing all literature, but a large collection of articles based on systematic search. Using citations as measures of article impact is not ideal, yet it remains to be the most followed practice in the literature. To compensate for such shortcoming, we used structural topic modeling to gather all relevant topics and insights from the literature. One should not expect that synthesizing a few thousands of papers in a single article can be exhaustive, comprehensive, or complete. Nonetheless, our results should be viewed as a summary of the “important” take-home messages from these articles. Bibliometrics methods have known deficiencies such as over-reliance on metrics and skewed quantification of research which we tried to avoid in our article by combining several methods. The recency of the pandemic does not allow an accurate estimation of the impact of research or a temporal timeline and therefore, our estimation of such aspects remains to be verified in future research. Last, relying on a single database may have missed some articles that are not indexed in Scopus. Nevertheless, we had to choose one database to avoid erroneous mixing of citation counts between databases, and we selected Scopus since it has a wide coverage. Another limitation for our study is reliance on a database with poor selection of articles from the global south, a problem that all databases suffer from.

## Conclusions

This work provides synthesis of COVID-19 research published by the educational community. A combination of quantitative analysis of research productivity with pandemic statistics, structural topic modeling and qualitative synthesis of papers with most attention from the educational community was used. A large volume of knowledge has been produced in education over the past couple of years that addressed various aspects of the pandemic, the majority of which had been published in open access journals, and few were in well-established publication outlets. From all papers that were taken into account, three main groups of topics were identified: (i) topics related to education in general, (ii) topics dealing with migration to online education, and (iii) diverse topics, e.g., perceptions, inclusion, medical education, engagement and motivation, well-being, and equality. A deeper analysis of the most cited papers revealed that problem understanding was the dominating theme of papers, followed by challenges, impact, guidance, online migration and tools and resources. While the conducted analysis may not be viewed as all encompassing, as some papers may have been missed by using one database, it does give an important synthesis of the findings in a large volume of knowledge as the insights were drawn from multiple perspectives and using different methods.

## Data Availability

The data in this manuscript are available with reasonable request given the licenser approval.
